# Lack of MERS Coronavirus but Prevalence of Influenza Virus in French Pilgrims after 2013 Hajj

**DOI:** 10.3201/eid2004.131708

**Published:** 2014-04

**Authors:** Philippe Gautret, Rémi Charrel, Samir Benkouiten, Khadidja Belhouchat, Antoine Nougairede, Tassadit Drali, Nicolas Salez, Ziad A. Memish, Malak al Masri, Jean-Christophe Lagier, Matthieu Million, Didier Raoult, Philippe Brouqui, Philippe Parola

**Affiliations:** Aix Marseille Université, Marseille, France (P. Gautret, R. Charrel, S. Benkouiten, A. Nougairde, N. Salez, J.-C. Lagier, M. Million, D. Raoult, P. Brouqui, P. Parola);; Institut Hospitalo-Universitaire Méditerranée Infection, Marseille (P. Gautret, R. Charrel, S. Benkouiten, K. Belhouchat, A. Nougairede, T. Drali, N. Salez, J.-C. Lagier, M. Million, D. Raoult, P. Brouqui, P. Parola);; Public Health Directorate, Saudi Ministry of Health, Riyadh, Kingdom of Saudi Arabia (Z.A. Memish, M. al Masri);; College of Medicine, Alfaisal University, Riyadh (A. Memish)

**Keywords:** Hajj, Middle East respiratory syndrome coronavirus, MERS-CoV, influenza, respiratory infections, France, Saudi Arabia, viruses

**To the Editor:** Saudi Arabia has reported the highest number of Middle East respiratory syndrome coronavirus (MERS-CoV) cases since the virus first emerged in 2012, with >127 confirmed cases and a case-fatality rate of 42%, as of November 2013 (*1*). Global attention has focused on the potential for spread of MERS-CoV after the Hajj pilgrimage during which Muslims from 180 countries converge in Mecca, Saudi Arabia. Such pilgrims have a high risk for respiratory tract infections because of severe overcrowding. The International Health Regulations Emergency Committee advised all countries (particularly those with returning pilgrims) to strengthen their surveillance capacities and ensure robust reporting of any identified cases (*2*).

We report the results of a prospective cohort study conducted in Saudi Arabia in October 2013. Participants in the survey were adult Hajj pilgrims who traveled together in a group (through 1 travel agency in Marseille, France) from October 3 through October 24, 2013. Pilgrims were included in the study on a voluntary basis and were asked to sign a written consent form. All pilgrims received advice about individual prevention measures against respiratory tract infection before departing, and follow-up was conducted during the journey by a medical doctor who systematically documented travel-associated diseases. Nasal swab specimens were obtained just before the pilgrims left Saudi Arabia, frozen <48 hours after sampling, and processed (*3*,*4*). Each sample was tested for MERS-CoV (upE and ORF1a genes) (*5*,*6*) and influenza A, B (*7*), and A/2009/H1N1 viruses (*8*) by real-time reverse transcription PCR. The protocol was approved by our Institutional Review Board (July 23, 2013; reference no. 2013-A00961–44) and by the Saudi Ministry of Health ethics committee. 

On departure from France, the study comprised 129 pilgrims. Their mean age was 61.7 years (range 34–85 years), and the male/female ratio was 0.7:1. Sixty-eight (52.7%) pilgrims reported having a chronic disease, including hypertension (43 [33.3%]), diabetes (34 [26.4%]), chronic cardiac disease (11 [8.5%]), and chronic respiratory disease (5 [3.9%]). Forty-six (35.7%) pilgrims reported receiving influenza vaccination in 2012; none had been vaccinated in 2013 before the Hajj because the vaccine was not yet available in France.

Clinical data were available for 129 persons: 117 (90.7%) had respiratory symptoms while in Saudi Arabia, including cough (112 [86.8%]) and sore throat (107 [82.9%]); 64 (49.6%) reported fever, and 61 (47.3%) had conditions that met the criteria for influenza-like illness (ILI; i.e., the association of cough, sore throat, and subjective fever) (Figure) (*4*). One patient was hospitalized during travel (undocumented pneumonia). Nasal swab specimens were obtained from 129 pilgrims on October 23, 2013 (week 43), 1 day before pilgrims left Saudi Arabia for France; 90 (69.8%) pilgrims were still symptomatic. All PCRs were negative for MERS-CoV.

**Figure:**
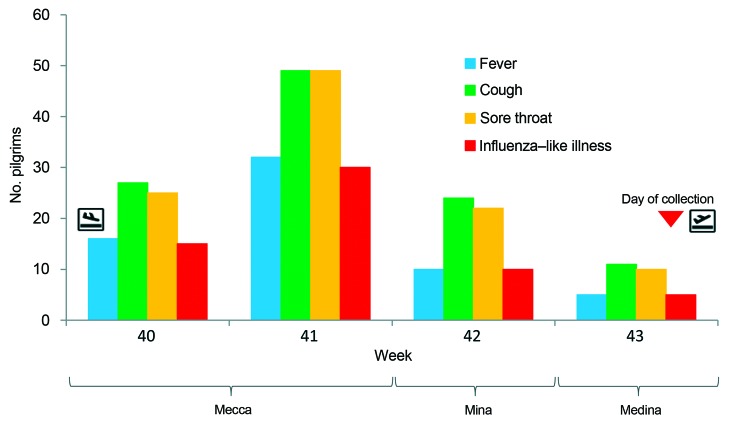
Onset of respiratory symptoms by week, reported by 129 Hajj pilgrims from France during their stay in Saudi Arabia, October 2013.

Eight pilgrims tested positive for influenza A(H3N2), 1 for influenza A(H1N1), and 1 for influenza B virus. No dual infections were reported. 70 (54.3%) pilgrims were seen 3–5 weeks after they returned to France, and the remaining were lost to follow-up. Fifty-five (78.6%) had experienced respiratory symptoms since their return, including cough (50 [71.4%]) and sore throat (14 [20.0%]); 12 (17.1%) reported fever, and illness in 5 (7.1%) pilgrims met the criteria for ILI. The 10 pilgrims who had positive test results for influenza virus on return had cleared their infection; only 1 additional sample was positive (for influenza A[H1N1]).

Our results support data obtained from a similar cohort in 2012 that showed a lack of nasal carriage of MERS-CoV among Hajj pilgrims from France (*3*). However, a higher prevalence of influenza virus (7.8%) was observed in nasal swab specimens in 2013 than in 2012 when 2 (3.2%) cases of influenza B virus infection were detected and no case of influenza A virus infection was detected among 162 pilgrims returning from the Hajj (*4*).

The estimated incidence of ILI in France during week 43 was 27 per 100,000 inhabitants, far below the epidemic threshold (126/100,000) with few sporadic cases of influenza A virus infection reported in some regions in France (www.grog.org/bullhebdo_pdf/bull_grog_43-2013.pdf). No case was reported in the Marseille area (http://websenti.u707.jussieu.fr/sentiweb). The high prevalence of respiratory symptoms in our cohort probably reflects the close surveillance performed and is consistent with 2012 results (*3*,*4*).

In Marseille, all patients with suspected MERS-CoV infection are referred to the Institut Hospitalo-Universitaire Méditerranée Infection. As of November 8, 2013, of the 14 first returning patients hospitalized for respiratory symptoms and screened for MERS-CoV and other pathogens, including influenza, 4 were infected with influenza A(H3N2), 4 with influenza A(H1N1), and 1 with influenza B virus. All samples tested negative for MERS-CoV.

Our preliminary results indicate that pilgrims from France returning from the 2013 Hajj were free of MERS-CoV but that a proportion were infected with influenza viruses and may represent a potential for early introduction of influenza in southern France. This proportion may have been underestimated because screening was performed at the end of the study period when some infections had cleared. Influenza vaccination should be a priority for pilgrims attending the Hajj (*9*,*10*).
